# Structural and Diffusion Property Alterations in Unaffected Siblings of Patients with Obsessive-Compulsive Disorder

**DOI:** 10.1371/journal.pone.0085663

**Published:** 2014-01-28

**Authors:** Ziwen Peng, Feng Shi, Changzheng Shi, Guodong Miao, Qiong Yang, Wei Gao, Jason J. Wolff, Raymond C. K. Chan, Dinggang Shen

**Affiliations:** 1 Department of Radiology and BRIC, University of North Carolina at Chapel Hill, North Carolina, United States of America; 2 Department of Psychology, South China Normal University, Guangzhou, China; 3 Medical Imaging Center, The First Affiliated Hospital of Jinan University, Guangzhou, China; 4 Guangzhou Psychiatry Hospital, Guangzhou, China; 5 Carolina Institute for Developmental Disabilities and the Department of Psychiatry, University of North Carolina at Chapel Hill, North Carolina, United States of America; 6 Neuropsychology and Applied Cognitive Neuroscience Laboratory, Key Laboratory of Mental Health, Institute of Psychology, Chinese Academy of Sciences, Beijing, China; 7 Department of Brain and Cognitive Engineering, Korea University, Korea; University of Ulm, Germany

## Abstract

Disrupted white matter integrity and abnormal cortical thickness are widely reported in the pathophysiology of obsessive-compulsive disorder (OCD). However, the relationship between alterations in white matter connectivity and cortical thickness in OCD is unclear. In addition, the heritability of this relationship is poorly understood. To investigate the relationship of white matter microstructure with cortical thickness, we measure fractional anisotropy (FA) of white matter in 30 OCD patients, 19 unaffected siblings and 30 matched healthy controls. Then, we take those regions of significantly altered FA in OCD patients compared with healthy controls to perform fiber tracking. Next, we calculate the fiber quantity in the same tracts. Lastly, we compare cortical thickness in the target regions of those tracts. Patients with OCD exhibited decreased FA in cingulum, arcuate fibers near the superior parietal lobule, inferior longitudinal fasciculus near the right superior temporal gyrus and uncinate fasciculus. Siblings showed reduced FA in arcuate fibers near the superior parietal lobule and anterior limb of internal capsule. Significant reductions in both fiber quantities and cortical thickness in OCD patients and their unaffected siblings were also observed in the projected brain areas when using the arcuate fibers near the left superior parietal lobule as the starting points. Reduced FA in the left superior parietal lobule was observed not only in patients with OCD but also in their unaffected siblings. Originated from the superior parietal lobule, the number of fibers was also found to be decreased and the corresponding cortical regions were thinner relative to controls. The linkage between disrupted white matter integrity and the abnormal cortical thickness may be a vulnerability marker for OCD.

## Introduction

Obsessive-compulsive disorder (OCD) is a chronically debilitating psychiatric disorder with a prevalance of approximately 2% [1]. The disorder is characterized by two symptom domains: *obsessions* (intrusive, recurrent thoughts, ideas or images) and *compulsions* (repetitive or compulsive behaviors). OCD is frequently familial, and first-degree relatives of individuals with OCD have a fivefold increased risk of developing the disorder [2], [3]. At present, there is relatively little known about the pathophysiology of OCD. Multi-modal neuroimaging studies of OCD have the potential to significantly advance our understanding of underlying neurobiology and inform treatment strategies.

Alterations in brain structures as well as disrupted connectivity in cortico-striato-thalamo-cortical circuitry have been implicated as pathophysiological mechanisms of OCD [4]. Several structural magnetic resonance imaging (MRI) studies, employing voxel-based morphometry (VBM), support this model [5], [6], [7], [8], [9]. For instance, Valente *et al.* reported that patients with OCD showed increased grey matter in the left posterior orbitofrontal cortex, anterior insula, bilateral parahippocampal gyrus, and right fusiform gyrus, and decreased grey matter in the left anterior cingulate gyrus and medial frontal gyrus, compared with healthy controls [6]. More recent studies have indicated that these grey matter alterations are accompanied by morphometric alterations in surface structure [10], [11], [12], [13]. For example, patients with OCD exhibit reduced cortical thickness in the left ventral cortex compared with matched controls, including the orbitofrontal cortex, inferior frontal gyrus, precentral gyrus, middle frontal gyrus, superior temporal gyrus, parahippocampal gyrus, and lingual gyrus [10]. Although many studies of grey matter provide support for the role of cortico-striato-thalamo-cortical circuitry, other brain regions outside of the classical circuit have been hypothesized to play a key role in the neurobiology of the disorder, such as the parietal regions [6], [7], [12], [13].

In addition to findings of atypical grey matter volumes and cortical thickness, there is increasing evidence of structural aberrations in white matter within OCD's classical neuroanatomical circuits [14], [15], [16], [17]. Using diffusion tensor imaging (DTI), Mori *et al.* reported that patients with OCD had significantly lower fractional anisotropy (FA), an indicator of the white matter microstructural integrity, in the bilateral anterior cingulate gyrus, parietal lobe, right posterior cingulate gyrus, and left lingual gyrus using VBM [18]. They also found that lower FA values in the parietal lobe were related with symptom severity in patients with OCD, which strongly suggests that parietal lobe connections may be dimensionally implicated in the pathophysiology of OCD [14]. Moreover, using tract-based spatial statistics (TBSS), Jayarajan and colleagues further found that widespread white matter abnormalities associated with OCD not only in cortico-striato-thalamo-cortical circuit but also in parietal regions, lateral prefrontal cortex, and limbic system when compared to healthy controls [15].

Of note, structural changes in grey and white matter have also been detected in high-risk subjects, which indicates that morphometric abnormalities may exist prior to illness onset and might even increase the risk for the subsequent emergence of clinical symptoms [19], [20], [21]. For example, Menzies *et al.* found that patients with OCD and their unaffected first-degree relatives both had reduced FA in the right inferior parietal lobe and increased FA in the right medial frontal lobe, indicating that these white matter abnormalities may be an endophenotype for OCD [19]. In addition, according to mechanical models of brain development, cortical gyrification and cortical thickness are considered to result from fiber connectivity exerted between connected brain regions [22], [23]. Consequently, early white matter disruption, which is a potential pathogenic mechanism of OCD, should be accompanied by the corresponding alterations in gyrification and cortical thickness starting early in development. Recently, several studies found that abnormal white matter development was associated with aberrant cortical thickness [24], [25], [26]. For example, Koch *et al.* found a significant linkage between disrupted white matter (in the superior temporal cortex) and reduced cortical thickness (in the posterior cingulate cortex, PCC) in patients with schizophrenia [24]. Franc and colleagues found that decreased FA in the optic radiations and posterior corona radiata were associated with lower average cortical thickness in individuals with type 1 diabetes using fiber tract projection analysis [25]. However, to our best knowledge, no reported study has investigated the potential linkage between disruptions in white matter and alterations in cortical thickness in patients with OCD.

Due to their high risk of developing OCD, unaffected siblings of patients with the disorder can provide rich genetic information for OCD research, thus helping disentangle the state and trait markers of the illness. Consequently, an important step towards investigating how combined alterations of white matter and cortical thickness may serve as a putative biological marker is to examine these alterations in the unaffected siblings of individuals with OCD.

The goal of the present study is to investigate two hypotheses: (1) whether there exists a potential linkage between disruptions in white matter connectivity and alterations in cortical thickness in patients with OCD; and (2) whether similar abnormalities are evident in unaffected siblings, thus representing a potential biomarker of increased risk for OCD. Specifically, in order to detect the correlation between abnormal white matter and abnormal cortical thickness, brain regions with significant FA differences between individuals with OCD and healthy controls were used as starting points for fiber tracking. Cortical thickness in regions implicated by these tracts, as well as the number of fibers connecting cortical regions, can be compared between groups of individuals with OCD, their siblings, and healthy controls. We expect that disrupted white matter integrity should go along with reduced cortical thickness in individuals with OCD, and, given the high heritability of OCD, similar abnormalities will be present in their unaffected siblings.

## Materials and Methods

### Subjects

A total of 30 adult outpatient subjects with a DSM-IV diagnosis of OCD, 19 unaffected full siblings, and 30 healthy subjects were included in this study (see Table 1 for sample characteristics). All subjects were right-handed. Subjects with OCD and their unaffected siblings were recruited through Guangzhou Psychiatry Hospital, in China, and healthy subjects were recruited by community and internet advertisements. Each paired patient-sibling came from one family, and these are nineteen separate families. All subjects with OCD were interviewed by a licensed psychiatrist (Z.W.P) and diagnosed using the Structured Clinical Interview (SCID) [27]. Diagnoses were confirmed by two independent clinical psychiatrists (G.D.M and Q.Y). All outpatient subjects had a primary diagnosis of OCD. Five of these subjects had comorbid major depressive disorder (3 recurrent and 2 with a single episode, without psychotic features), 3 had social phobia, 1 had an eating disorder, and all others had OCD as their sole diagnosis. All patients were received with stable drug treatment for at least 4 weeks, and seventeen patients were mediated with selective serotonin reuptake inhibitors (SSRI) as a single drug, and the other thirteen patients were treated with additional medications or antidepressants of other classes (with the details provided in the Table S1).

**Table 1:** Demographic and clinical characteristics for participants.

	OCD (N = 30)	SIB (N = 19)	CON (N = 30)	Analysis	
Characteristic	Mean (SD)	Mean (SD)	Mean (SD)	F(df = 2, 76)	p-value
Age (years)	28.0 (6.8)	28.6 (8.5)	27.3 (8.2)	0.17	0.84
Male/Female	21/9	9/10	22/8		0.15
Education (years)	13.7 (3.0)	14.6 (2.4)	13.3 (3.7)	1.10	0.34
IQ estimate	104.2 (16.7)	104.7 (14.8)	107.2 (15.7)	0.30	0.74
Age at onset of OCD (years)	19.5 (5.5)				
Duration of illness (years)	8.6 (5.5)				
Y-BOCS (total)	27.7 (6.2)	3.8 (3.7)	2.0 (2.7)	279.80	< 0.01
Y-BOCS (obsessions)	15.7 (3.5)	1.7 (1.9)	0.9 (1.2)	315.46	< 0.01
Y-BOCS (compulsions)	12.0 (4.9)	2.2 (2.2)	1.1 (1.9)	88.80	< 0.01
OCI-R	21.9 (12.2)	9.0 (6.6)	10.3 (9.3)	13.73	< 0.01
BDI	17.0 (13.7)	3.5 (7.4)	7.2 (8.0)	11.54	< 0.01
STAI (state)	49.9 (17.7)	29.1 (15.1)	34.5 (18.1)	10.08	< 0.01
STAI (trait)	52.1 (16.8)	29.4 (17.6)	34.1 (17.8)	12.44	< 0.01

Abbreviations: BDI, Beck Depression Inventory; CON, healthy control subjects; OCD, patients with Obsessive-Compulsive Disorder; OCI-R, Obsessive-Compulsive Inventory-Revised; SD, Standard Deviation; SIB, siblings of individuals with OCD; STAI, State-Trait Anxiety Inventory; Y-BOCS, Yale-Brown Obsessive-Compulsive Scale.

Unaffected siblings and healthy subjects were interviewed by the SCID-I/NP [28]. Both groups had no history of psychiatric illness. Moreover, healthy comparison subjects reported no history of psychiatric illness within their third-degree relatives. Exclusion criteria included psychosis, bipolar disorder, neurological disorder (including tic disorders), head injury, serious medical condition, history of drug or alcohol addiction, and cardiac pacemakers or other metallic implants.

All procedures were approved by the ethics committee of Guangzhou Psychiatry Hospital, China, and written informed consent was obtained from all participants.

### Clinical assessments

Obsessive-compulsive (OC) symptom severity was evaluated using the Yale-Brown Obsessive-Compulsive Scale (Y-BOCS) [29]. The Obsessive-Compulsive Inventory-Revised (OCI-R) was used to assess OC symptom substyles [30], [31]. Symptoms of depression and anxiety were quantified using the Beck Depression Inventory (BDI) [32] and the State-Trait Anxiety Inventory (STAI) [33]. The Annett Handedness Inventory was used to measure handedness [34]. Estimated IQ was assessed with the short form of the Chinese version of the Wechsler Adult Intelligence Scale-Revised (WAIS-R), including four subscales: information, arithmetic, similarity, and digit span [35], and subjects with a total standard score of less than 80 were excluded.

### MRI procedures

After clinical interview and assessment, participants underwent MRI examination. The MRI data were acquired on a Signa HDe 1.5-T GE scanner (GE Medical Systems, Milwaukee, WI, U.S.A.) equipped with an 8-channel phased-array head coil at the First Affiliated Hospital of Jinan University. Diffusion-weighted images were obtained using echo-planar imaging (TR = 10000 ms; TE = 95.4 ms; flip angle = 90°; FOV = 120×120 mm; matrix = 128×128; slice thickness = 4.0 mm; Number of axial slices = 30). Diffusion-sensitizing gradient encoding was applied in 25 different directions with a diffusion-weighing factor of b = 1000 s/mm^2^ and one without diffusion gradient (b = 0) image. Images were acquired parallel to the anterior-posterior commissure. A high-resolution T1-weighted anatomical image was acquired by using a 3-dimensional fast spoiled gradient recalled (FSPGR) sequence with 128 contiguous slices (TR = 8 ms; TE = 1.7 ms; flip angle = 20°; FOV = 240 mm×240 mm; matrix = 256×256; slice thickness = 1.0 mm). Magnetic field uniform was reached before each scanning. All the images were visually inspected for clinical abnormalities and common artifacts by a radiologist (C.Z. S.) prior to analysis.

### Imaging analysis

#### DTI pre-processing and FA group analysis

First, the DTI data were corrected for motion and eddy current effects by applying an affine alignment of the diffusion-weighted images to the b0 image using the FSL software package (http://www.fmrib.ox.ac.uk/fsl) [36]. Second, diffusion tensors at each voxel were constructed using a weighted-least-squares estimation algorithm [37]. and FA images were computed. Third, the resulting FA images were transformed into Montreal Neurological Institute (MNI) standard space for voxelwise group comparison with SPM8 (http://www.fil.ion.ucl.ac.uk.spm), including the following steps: a) each subject's b0 image was coregistered to T1 image, and the same coregistration parameters were applied to the FA map; b) each subject's T1 image was then nonlinearly normalized to the SPM T1 template, and the same normalization parameters were applied to the respective aligned FA maps; c) The final normalized FA map was further smoothed with a 6-mm full-width at half-maximum (FWHM) isotropic Gaussian kernel. A white matter mask was defined by binarizing the SPM white matter template to restrict the search volume for analysis during the corrections for multiple comparisons. FA maps of all subjects were then put into a general linear model (GLM) to remove the effects of age, gender, and mean FA of whole brain. Statistical analyses were conducted in SPM8. Statistical significance of FA between groups was finally evaluated by two-sample t-test, and the significance level was set at *p*<0.05, corrected for multiple comparisons (false discovery rate, FDR), with a minimum cluster size of 200 voxels.

### ROI-based fiber count analysis

Regions with significantly differed FA between OCD patients and healthy controls were selected as regions of interest (ROIs) in MNI template space. We then mapped each ROI back to the native space of each subject, and use it as the starting point for streamline fiber tracking by using ExploreDTI toolbox [38]. The following parameters were used for tractography: minimal seed-point FA = 0.25; minimal allowed FA = 0.20; maximal turning angle = 70°; minimal fiber length = 20 mm; and maximal fiber length = 400 mm. The resulting fiber tracts were reviewed to ensure their correctness by using ParaView (Kitware, http://www.paraview.org/), and the number of fiber tracts passing through each ROI was calculated. The obtained ROI-based fiber count was compared between each pair of groups after regressing out the effects of age, gender, and total fiber count of each subject.

### Fiber-projected cortical thickness analyses

Individual T1 MRI images were processed using the FreeSurfer software package [39]. Briefly, the T1 image for each subject was skull-stripped, tissue-segmented, and normalized to a common Talairach space. The white matter surface was first generated and then deformed from the boundary of gray matter to the pial surface. Next, the nearest vertex in the cortical surface where each axonal fiber terminated was chosen, and further the mean cortical thickness across all chosen cortices in each ROI (used as starting points for fiber tracking) was computed [40]. The obtained ROI-based mean cortical thickness was compared between each pair of groups after regressing out the effects of age, gender, and mean whole-brain cortical thickness of each subject.

### Regression analysis with clinical variables

In order to examine the relationship between clinical variables and FA values, cortical thickness, and the number of fibers in OCD patient group, we extracted the mean value of the corresponding morphometric measures from each ROI. A linear regression was carried out with all morphometric measures entered simultaneously as predictors for each clinical variable (i.e., duration of illness, age at onset of OCD, YBOCS, BDI and OCI-R). Age, gender and IQ were entered as covariates in this regression.

## Results

### Demographic data

Demographic and clinical data are shown in Table 1. The three groups were well-matched in age, IQ, gender, years of education and handness. There were significant differences in clinical measures (Y-BOCS, OCI-R, BDI, STAI) between the three groups. Post hoc tests found that patients with OCD had higher scores than healthy controls or siblings in total scores for the Y-BOCS, OCI-R, BDI, and STAI. Siblings did not differ from healthy controls in any of these measures.

### FA group analysis

**Figure 1:**
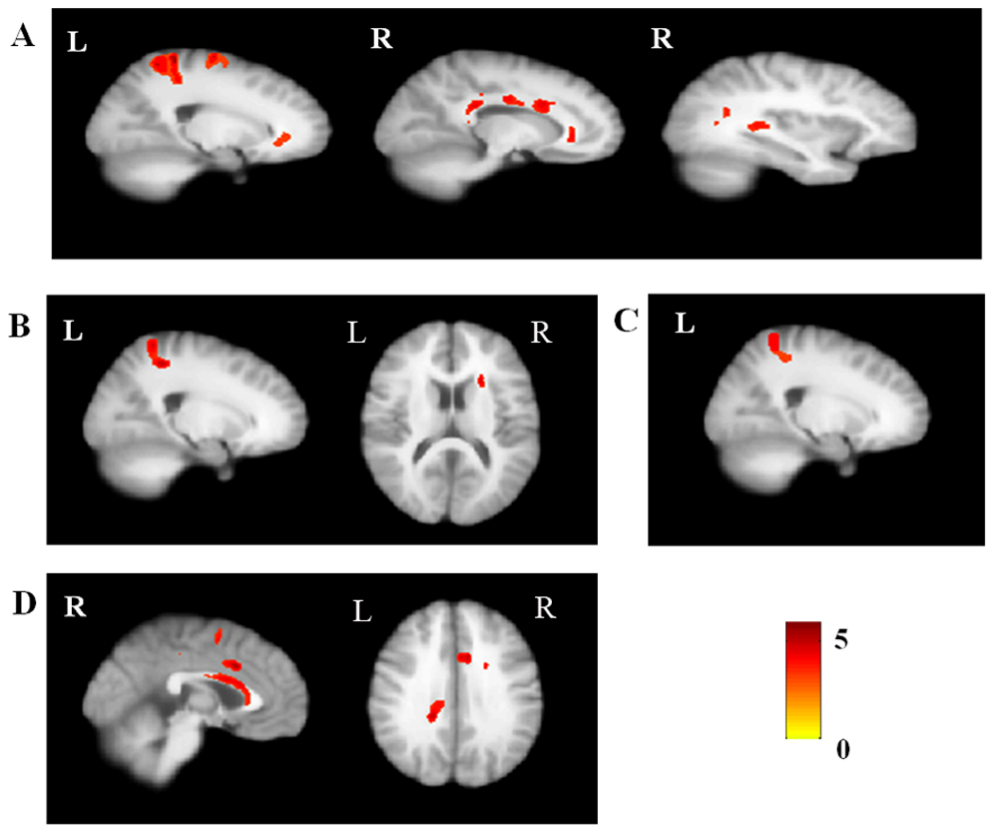
Regions showing significant fractional anisotropy (FA) differences between patients with OCD, their unaffected siblings, and healthy controls. (A) patients with OCD<healthy controls; (B) Siblings<healthy controls; (C) The common regions in which patients with OCD and unaffected siblings both had significant FA differences compared to the healthy controls; (D) Patients with OCD<siblings.

Group comparisons showed a pattern of reduced FA in patients with OCD compared with healthy controls, which includes the cingulum, arcuate fibers near the superior parietal lobule, inferior longitudinal fasciculus (ILF) near the right superior temporal gyrus and uncinate fasciculus. Relative to the unaffected siblings, patients with OCD exhibited decreased FA in the cingulum and superior longitudinal fasciculus (SLF), while no increased FA was found. Compared with healthy controls, unaffected siblings showed the reduced FA in arcuate fibers near the superior parietal lobule and anterior limb of internal capsule. Patients with OCD and their unaffected siblings both showed the decreased FA in arcuate fibers near the superior parietal lobule. Figure 1 shows these group-level comparisons.

### Fiber tracking analysis

**Figure 2:**
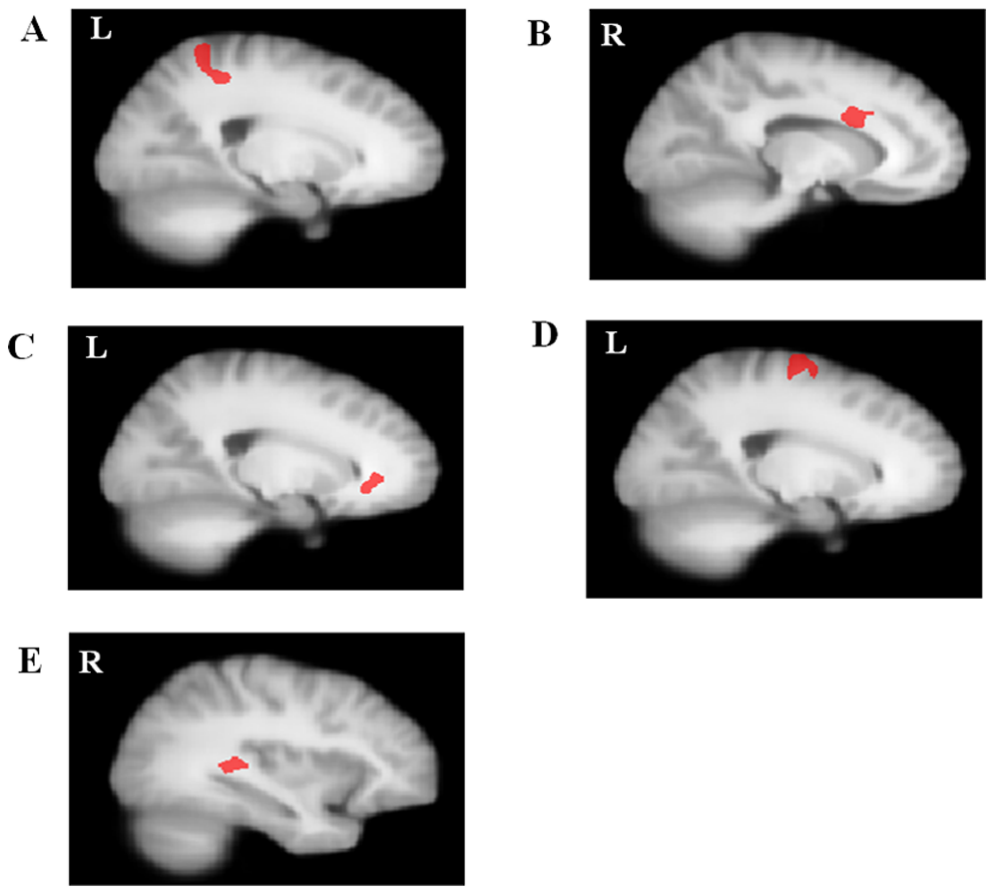
Regions used as starting points for fiber tractography analysis. (A) Arcuate fibers near left parietal lobe; (B) Cingulum near right corpus callosum-body; (C) Cingulum near left corpus callosum-genu; (D) Arcuate fibers near left precentral gyurs; (E) Inferior longitudinal fasciculus near right superior temporal gyrus.

**Figure 3:**
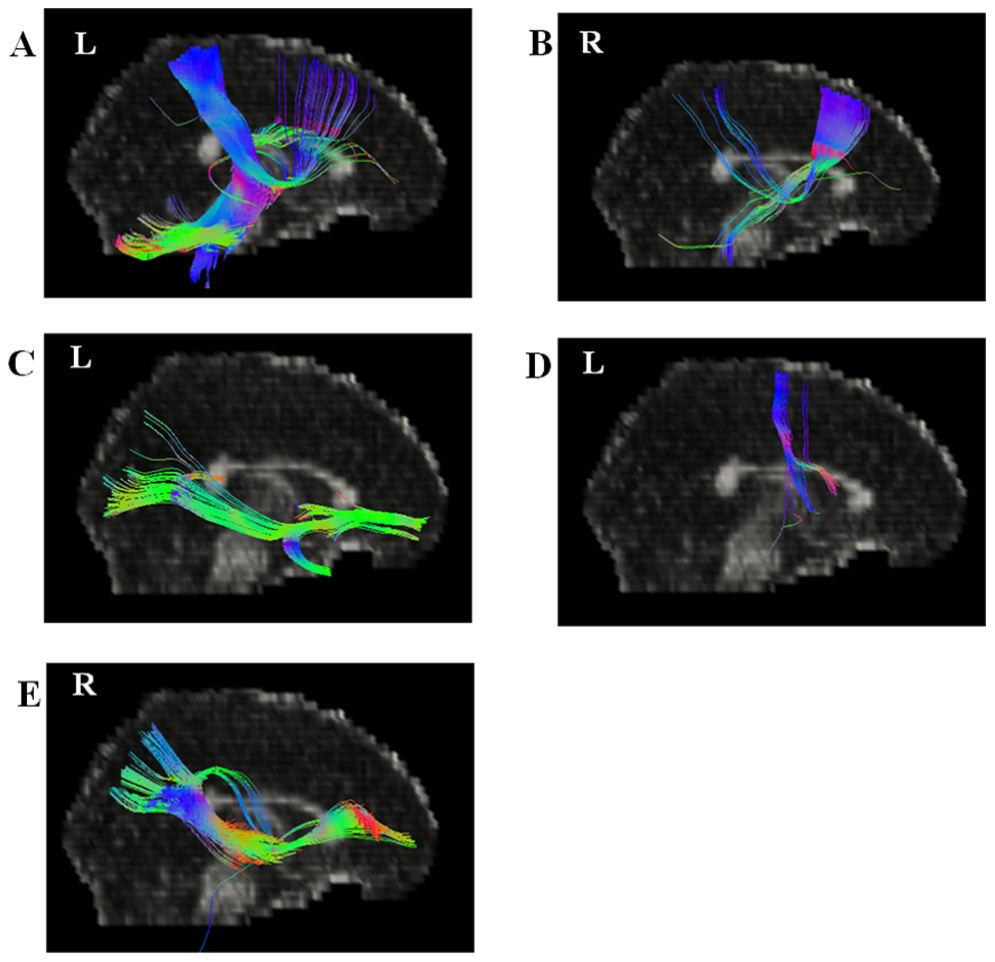
Fiber connectivity maps from starting points in all subjects. (A) Arcuate fibers near left parietal lobe; (B) Cingulum near right corpus callosum-body; (C) Cingulum near left corpus callosum-genu; (D) Arcuate fibers near left precentral gyurs; (E) Inferior longitudinal fasciculus near right superior temporal gyrus.

**Figure 4:**
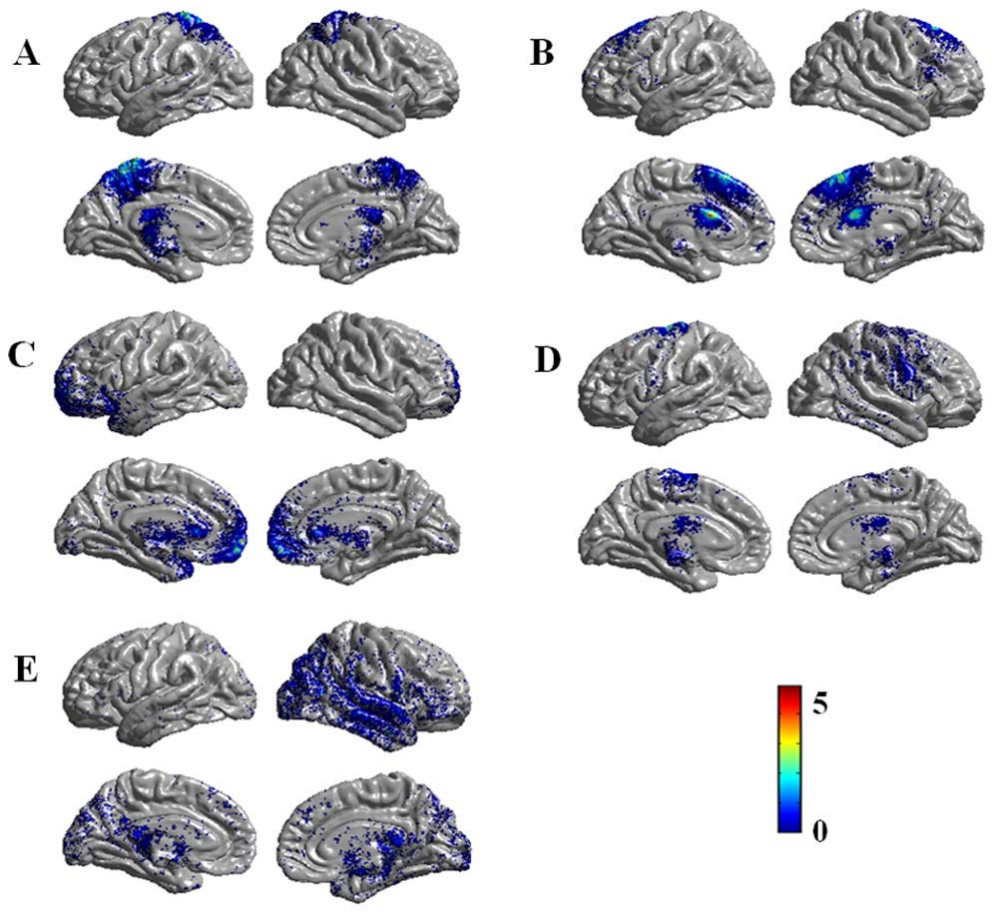
Projected cortical surface maps linked with starting points in all subjects. (A) Arcuate fibers near left parietal lobe; (B) Cingulum near right corpus callosum-body; (C) Cingulum near left corpus callosum-genu; (D) Arcuate fibers near left precentral gyurs; (E) Inferior longitudinal fasciculus near right superior temporal gyrus.

The present study was aimed to investigate the potential vulnerability markers for OCD, which means that these markers should exist in both OCD patients and their siblings [41]. Based on this hypothesis, we emphasized on the combined regions where OCD patients and their siblings both demonstrated abnormal FA compared with healthy controls, and these regions were postulated to be related with the disease. According to the DTI results, the cingulum near right body-CC and left genu-CC, the arcuate fibers near left precentral gyrus and left superior parietal lobule (the combined region in OCD vs Controls and Sibling vs Controls) and the ILF near right superior temporal gyrus were used as starting points for the fiber tracking analysis (see Fig. 2). Figure 3 shows the fiber tracking results from the five starting points in a mean atlas image, which was obtained by averaging all subjects' aligned images using tensor-based registration. Subsequent analysis determined which cortical regions had high connectivity with these starting points. Figure 4 and Table 2 shows cortical regions identified based on high connectivity with the five white matter starting points.

**Table 2:** Overview of the regions used for fiber tracking and their corresponding projected cortical regions.

	MNI coordinates			
Starting points for fiber tracking	*X*	*y*	*z*	Projected cortical regions
L arcuate fibers near superior parietal lobule	−38	−30	−34	Postcentral gyrus, precentral gyrus, superior parietal lobule, and paracentral lobule
R cingulum near corpus callosum-body	9	12	31	Superior frontal gyrus, and middle frontal gyrus
L cingulum near corpus callosum-genu	0	−33	29	OFC, ACC, and temporal pole
L arcuate fibers near precentral gyurs	−19	−14	73	Precentral gyrus, postcentral gyrus, and superior frontal gyrus
R ILF near superior temporal gyrus	50	0	1	Temporal lobe, occipital lobe, and middle frontal gyrus

ACC: Anterior cingulated cortex; ILF: inferior longitudinal fasciculus; L: left; OFC: Orbitofrontal cortex; R: right.

### Number of fibers within the high-connectivity cortical regions

**Figure 5:**
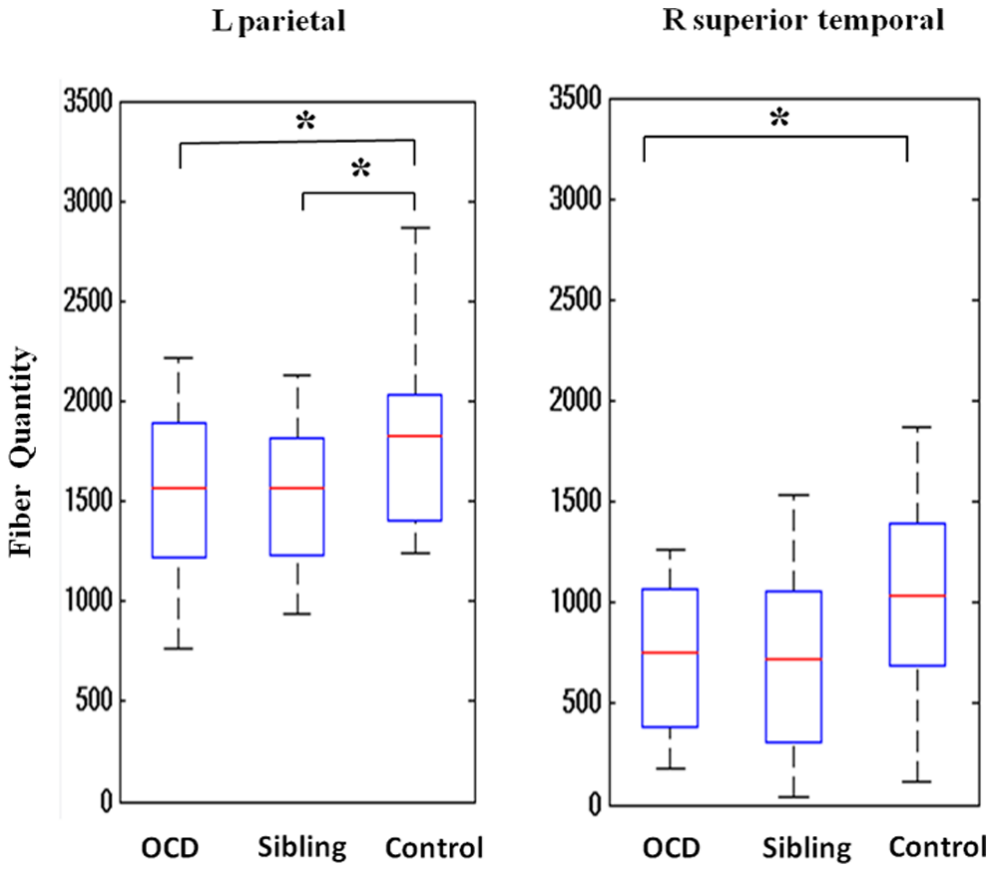
Significant group differences in fiber quantity in patients with OCD, their unaffected siblings, and healthy controls. Arcuate fibers near left superior parietal lobule (on the left panel) and inferior longitudinal fasciculus near right superior temporal gyrus (on the right panel) were used as starting points respectively. ^*^ *P*<0.05.

Compared with healthy controls, patients with OCD and their siblings both showed significantly decreased fiber count within the high-connectivity cortical regions measured using the arcuate fibers near superior parietal lobule as a starting point (P_OCD_ = 0.034, uncorrected; P_Siblings_ = 0.046, uncorrected). Patients with OCD also exhibited lower fiber counts in corresponding cortical regions compared to healthy controls using the ILF near right superior temporal gyrus as a starting point (P = 0.046, uncorrected). Figure 5 shows the details.

### Cortical thickness within the high-connectivity cortical regions

**Figure 6:**
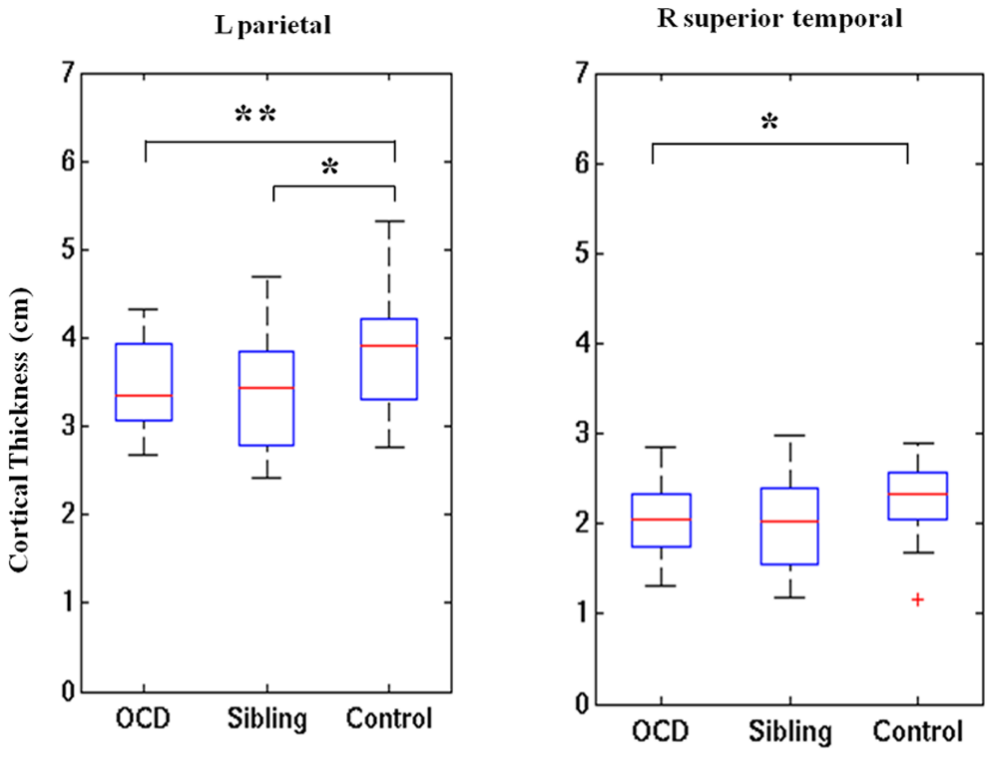
Significant group differences in cortical thickness in patients with OCD, their unaffected siblings, and healthy controls. Arcuate fibers near left superior parietal lobule (on the left panel) and inferior longitudinal fasciculus near right superior temporal gyrus (on the right panel) were used as starting points, respectively. ^*^ *P*<0.05. ^**^ *P*<0.01.

Subjects with OCD had significantly decreased cortical thickness compared to healthy controls within high-connectivity cortical regions when using the arcuate fibers near superior parietal lobule (P = 0.006, uncorrected) and the ILF near right superior temporal gyrus (P = 0.031, uncorrected) as starting points. NO areas of increased cortical thickness associated with OCD were found. Compared with healthy controls, unaffected siblings were similarly characterized by significantly less cortical thickness in the left superior parietal lobule (P = 0.023, uncorrected). No significant differences in cortical thickness were found between patients with OCD and their unaffected siblings. Cortical thickness data are presented in Figure 6. We also calculated the between-group difference of whole-brain cortical thickness within the three groups after regressing out the effects of age, gender and mean whole brain thickness (see Fig. S1). Some brain areas are overlapped with these areas projected by using left superior parietal lobule and the right superior temporal gyrus as starting points.

### Regression analysis with clinical variables

Our findings revealed that greater obsession subtotal scores on the Y-BOCS were correlated significantly with lower FA in the arcuate fibers near superior parietal lobule (P = 0.01, uncorrected). The value of projected cortical thickness using superior parietal lobule as starting point had a significant association with the total scores of OCI-R (P = 0.02, uncorrected). None of the other morphometric measures were significantly associated with these clinical variables (i.e., years of illness duration, age of onset, YBOCS, OCI-R and BDI). The relationship between whole-brain FA values and clinical variables and also the relationship between cortical thickness and clinical variables are shown in Fig. S2 and Fig. S3, respectively. In addition, the three imaging variables (FA, fiber quantity, and cortical thickness) were highly inter-correlated with each other, especially in the starting point of arcuate fibers near superior parietal lobule (see Table S2).

## Discussion

To the best of our knowledge, the current study is the first to investigate the combined relationship between white matter microstructure and cortical thickness in patients with OCD and their unaffected siblings. We found that patients with OCD exhibited reduced FA in the cingulum, arcuate fibers near the superior parietal lobule, the ILF near right superior temporal gyrus and uncinate fasciculus, compared with healthy controls. Similarly, siblings exhibited lower FA in the arcuate fibers near left superior parietal lobule than that observed in the healthy controls. Interestingly, relative to healthy controls, patients with OCD showed not only decreased fiber quantities, originated from the left superior parietal lobule seed, but also significantly thinner cortical thickness within corresponding cortical regions. Importantly, unaffected siblings exhibited similar fiber counts and cortical thickness abnormalities as patients with OCD. Overall, the current study indicates that the disruption of white matter in the left superior parietal lobule is accompanied by the reduced fiber quantities and cortical thickness within projection regions in both patients with OCD and their unaffected siblings, suggesting possible hereditary risk factors for OCD.

Our findings provide evidence of atypical structural neural circuitry in individuals with OCD. Specifically, we found decreased FA in the cingulum, arcuate fibers near the superior parietal lobule, the ILF near right superior temporal gyrus and uncinate fasciculus in patients with OCD, compared with healthy controls. These fiber bundles directly connect the brain regions within the cortico-striato-thalamo-cortical circuitry. For instance, the uncinate fasciculus is a prominent white matter tract connecting the OFC with the limbic system, such as hippocampus and amygdala in the temporal lobe [42], which are the core regions in the circuitry. Our results found that OCD patients exhibited abnormal white matter near the superior parietal lobule, which is consistent with previous studies [14], [19], [43]. In addition, first-degree relatives of individuals with OCD have also exhibited reduced FA in the parietal lobe, raising the possibility that this characteristic may be an endophenotype for OCD [19]. Intriguingly, our results indicated that white matter's alterations in the superior parietal lobule were linked to the corresponding alterations in cortical thickness, providing a deeper understanding of the pathophysiological mechanisms underlying white and grey matter deficits in OCD. Our results found that lower FA in the left superior parietal lobule was correlated with the severity of obsessive symptoms. Recently, a magnetic resonance spectroscopy study demonstrated that OCD patients had concentration changes of choline-containing compounds in the parietal white matter, but not in other regions of the brain. Furthermore, these changes were correlated with OCD symptom severity, which suggests the phospholipid abnormalities of myelinated axons in this region [43].

The left parietal lobe is associated with motor attention. Rushworth et al. found that subjects with impairment in left parietal lobe had a difficulty in shifting the focus of motor attention from one movement in a sequence to the text, while not in the right parietal lobe [44], which is core cognitive deficit for OCD and is suggested to be related with compulsive behaviors [45]. In the present study, decreased FA in superior parietal lobule white matter was associated with decreased cortical thickness in highly interconnected cortical regions (mainly postcentral gyrus, precuneus, PCC, paracentral lobule and sporadic prefrontal cortex, see Fig. 4A). The superior parietal lobule connects directly to the cingulated gyrus, the frontal lobe (precentral gyrus, postcentral gyrus, superior frontal gyrus, and inferior frontal gyrus), as well as the temporal lobe by the cingulum bundle [46], which is similar to the above projected cortical regions. These brain regions (DLPFC, postcentral gyrus, paracentral lobule, and thalamus) are highly linked with the superior parietal lobule and are mainly located in the indirect cortical-striatal-thalamic-cortical circuit, acting as a negative feedback loop for behavioral inhibition and switching, and thus integral to the core deficits associated with OCD [47].

Functionally, Stern *et al.* found that patients with OCD exhibited increased connectivity within the fronto-parietal network (FPN) compared with matched controls at the resting state [48]. Several brain regions were reported in their results, including the somatosensory and motor cortices, lateral frontal cortex, and the thalamus, all of which are the cortical regions implicated in the current study using fiber tracking analysis initiated in the parietal lobe and also connected with superior parietal lobe by cingulum bundle (see Fig. 4A). Recently, a lesion study reported that an OCD patient was free of OC symptoms immediately after infarction in the fronto-parietal cortex, implying that fronto-parietal cortex contributes to the pathogenesis of OCD [49].

Although functional connectivity is not a direct proxy for anatomic connectivity, it is possible that anatomic connectivity constrains neural activity fluctuations. Actually, coupled fluctuations were observed between many regions that possess anatomic connections [50]. Therefore, structural and functional interactions between the abnormal white matter in the superior parietal lobule and the alterations in cortical thickness may suggest that the reduced white matter integrity is closely associated with or may even cause grey matter alterations in the anatomically connected brain regions. Our results supported the hypothesis that there is a potential linkage between the disruptions in white matter connectivity and the alterations in cortical thickness in patients with OCD. A potential explanation for our finding is that the atypically developed white matter may result in impaired axonal transport, thus leading to inferior nutrient supply to the targeting neurons and finally anterograde and retrograde degeneration in neuron body [51]. Furthermore, white and grey matter loss is reversible and linked to improvement of OC symptoms, again implying a positive correlation between white and gray matter change [52]. The mechanism for the concurrent white and grey matter structural changes in OCD is still unclear. A recent genetic study found that OCD was associated with the OLIG2 gene, which is an essential regulator in the development of white matter and highly expressed in the cortico-striato-thalamo-cortical regions [53]. Those findings indicated that combined white and grey matter abnormalities may be related to the etiology of OCD. In addition, based on the tension-based theory of morphogenesis [22] that white matter is more susceptible to microvascular disease than cortical grey matter [54], it is plausible that white matter changes may be evident prior to grey matter alterations.

Importantly, abnormal white matter near the superior parietal lobule and decreased cortical thickness was also found in unaffected siblings, suggesting a possible endophenotype for OCD. Previous studies have separately provided evidence that white matter [19] and grey matter [21] alterations may be endophenotypes of OCD. However, no study has examined the correspondence between abnormal white matter and grey matter in OCD. Our findings provide new evidence that abnormal white matter near superior parietal lobule, significantly linked to grey matter alterations, may be a heriditary, familial characteristic of OCD. This suggests that abnormal white and grey matter are not only associated with the manifestation of OCD, but with familial risk as well. Conceptually, endophenotypes are quantitatively heritable traits that are abnormal in both probands and their relatives [41]. Therefore, brain differences apparent only in subjects with OCD, such as in the cingulum, ILF near right superior temporal gyrus and uncinate fasciculus along with specific white matter alterations, may be state characteristics of OCD. These imaging features could be used to classify OCD patients from the ordinary population as suggested by previous neuroimaging studies [55], [56], [57].

The current study has several potential limitations. First, all patients with OCD were being treated with psychotropic medications at the time of MRI examination, and the potential effect of these medications on FA, cortical thickness, and behavioral assessments cannot yet be determined. However, drug-naïve patients with OCD have been found to exhibit similar brain differences to those observed in the individuals with OCD and a history of drug treatment [58], [59]. Future research should directly investigate whether medication alters white matter microstructure and corresponding cortical thickness in patients with OCD. Second, the DTI acquisition sequence (25 directions) at 1.5T may not be characterized as cutting-edge. But the reliability of such sequence has been extensively evaluated by many previous studies [60], [61]. Third, using VBM analysis in DTI study may be not the up-to-date method. We selected the VBM method mainly because the abnormal white matter in the parietal lobe was found both in OCD patients and their first-degree relatives in a previous VBM-based study, which aimed to coincide with their method [19]. On the other hand, compared to tract-based analysis, voxel-based analysis has the advantage of keeping partial volume effects with gray matter and white matter from other bundles to a minimum [60], [62]. Fourth, the regions with the significantly-altered FA in OCD patients compared with healthy controls were selected to carry out fiber tracking analysis, which could cause the selection bias by the circular analysis [63]. Future studies are needed to use independent data for region selection and also use the selective analyses to prevent the possible circularity [64].

## Conclusions

In summary, our data provides substantial evidence that disrupted white matter microstructure was associated with decreased cortical thickness in corresponding cortical regions in patients with OCD and their unaffected siblings when using the white matter near superior parietal lobule as a starting point for tractography, suggesting that this feature may be an endophenotype representing increased genetic risk for OCD.

## Supporting Information

Figure S1 **Regions showing significant cortical thickness difference between patients with OCD, their unaffected siblings, and healthy controls.**
(ZIP)

Figure S2 **The regression analysis of whole-brain FA and clinical variables in OCD patient group.**
(ZIP)

Figure S3 **The regression analysis of cortical thickness and clinical variables in OCD patient group.**
(ZIP)

Table S1 **Treatment details of OCD patients.**
(ZIP)

Table S2 **Inter-correlations between the anatomical measurements.** The correlation coefficients between each pair of mean FA, fiber-quantity (FQ) and mean cortical-thickness (CT) values from each of the 5 ROIs are shown below.
(ZIP)
