# Supplementary material for: Structural and Diffusion Property Alterations in Unaffected Siblings of Patients with Obsessive-Compulsive Disorder
Source: PLoS One. 2014 Jan 28;9(1):e85663. doi: 10.1371/journal.pone.0085663 (PMC3904847; doi:10.1371/journal.pone.0085663)
Supplement: Figure S1 — Regions showing significant cortical thickness difference between patients with OCD, their unaffected siblings, and healthy controls. (ZIP) [file pone.0085663.s001.zip]

**Whole-brain cortical thickness analysis**

The cortical thickness analysis revealed a pattern of widespread cortical abnormality in both hemispheres in the OCD patient group. Compared with healthy controls, OCD patients showed thinner thickness in the dorsolateral prefrontal cortex (DLPFC), medial frontal cortex, temporal lobe, precentral gyrus, postcentral gyrus, lingual gyrus, and parahippocompal gyrus. Unaffected siblings exhibited decreased cortical thickness in the postcentral gyrus, occipital cortex, superior temporal gyrus, paracentral gyrus, and inferior frontal cortex (mainly in the pars triangularis), compared with healthy controls. Relative to the unaffected siblings, patients with OCD showed reduced cortical thickness in the DLPFC, paracentral gyrus, lingual gyrus, cingulated gyrus, and pars triangularis cortex. Figure S1 shows the detailed abnormal patterns.

**
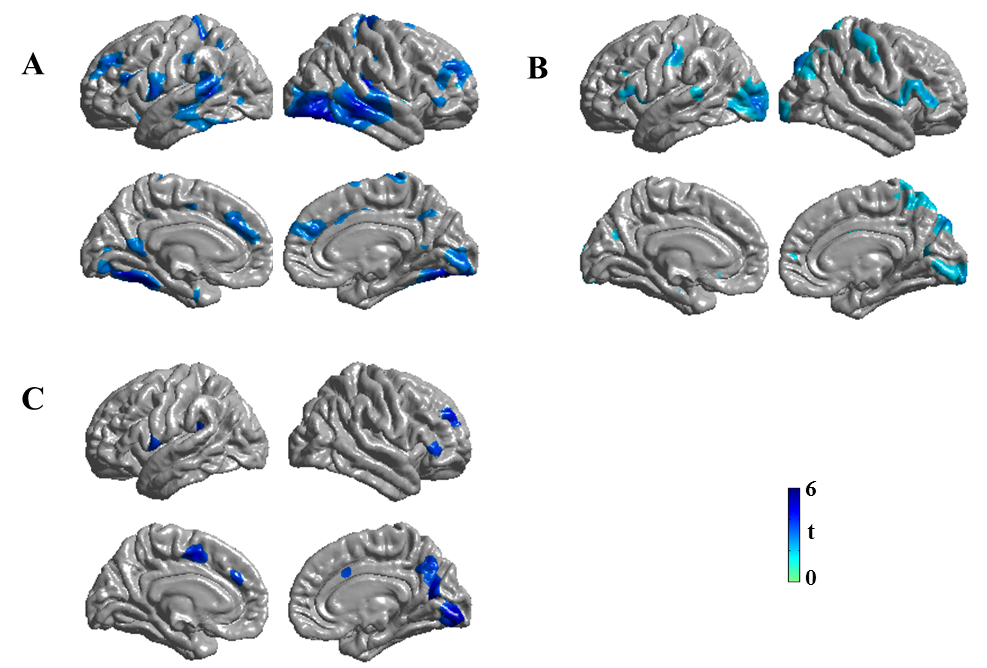
**

**Fig. S1.** Regions showing significant cortical thickness difference between patients with OCD, their unaffected siblings, and healthy controls. (A) Patients with OCD < healthy controls; (B) Siblings < healthy controls; (C) Patients with OCD < siblings. The significance level was set at *p* < 0.05 with FDR correction.
