# Supplementary material for: Structural and Diffusion Property Alterations in Unaffected Siblings of Patients with Obsessive-Compulsive Disorder
Source: PLoS One. 2014 Jan 28;9(1):e85663. doi: 10.1371/journal.pone.0085663 (PMC3904847; doi:10.1371/journal.pone.0085663)
Supplement: Figure S2 — The regression analysis of whole-brain FA and clinical variables in OCD patient group. (ZIP) [file pone.0085663.s002.zip]

**The regression analysis between whole brain FA and clinical variables in OCD patients group**

A linear regression was carried out by using FA value in each voxel as predictor for each clinical variable (YBOCS and OCI-R), and the significance level was set at *p* < 0.05 with FDR correction. Age, gender and IQ were entered as covariates in this regression. The details were showed in Fig. S2.


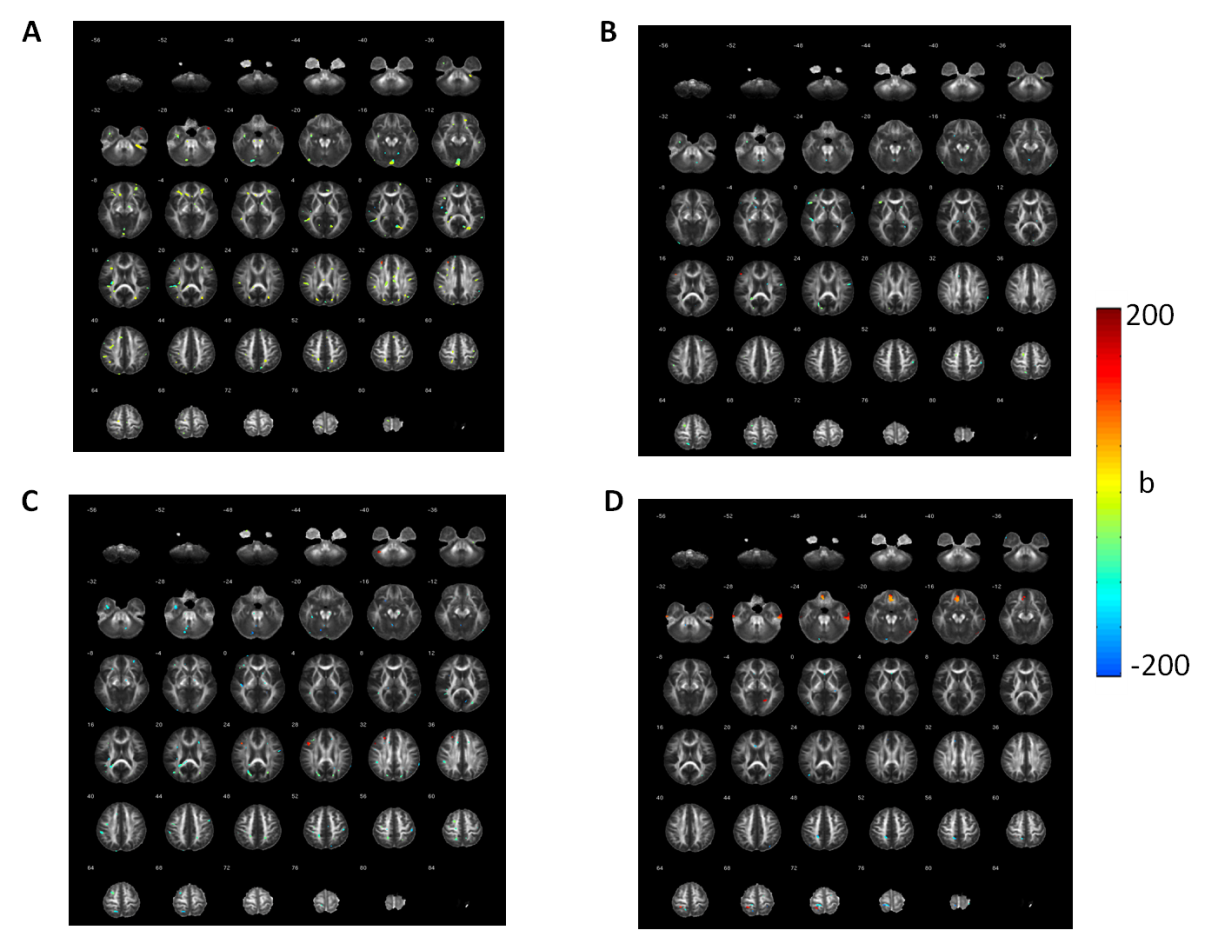


**Fig. S2.** The regression analysis of whole-brain FA and clinical variables in OCD patient group. Age, gender, and IQ are entered as covariates. Color indicates the regression coefficient of FA values with clinical scores. Subfigures denote the results on 4 different clinical scores: (A) Obsessive subtotal scores on the Y-BOCS; (B) Compulsive subtotal scores on the Y-BOCS; (C) Total scores of Y-BOCS; (D) Total scores of OCI-R.
