# Supplementary material for: Structural and Diffusion Property Alterations in Unaffected Siblings of Patients with Obsessive-Compulsive Disorder
Source: PLoS One. 2014 Jan 28;9(1):e85663. doi: 10.1371/journal.pone.0085663 (PMC3904847; doi:10.1371/journal.pone.0085663)
Supplement: Figure S3 — The regression analysis of cortical thickness and clinical variables in OCD patient group. (ZIP) [file pone.0085663.s003.zip]

**The regression analysis between whole brain cortical thickness and clinical variables in OCD patients group**


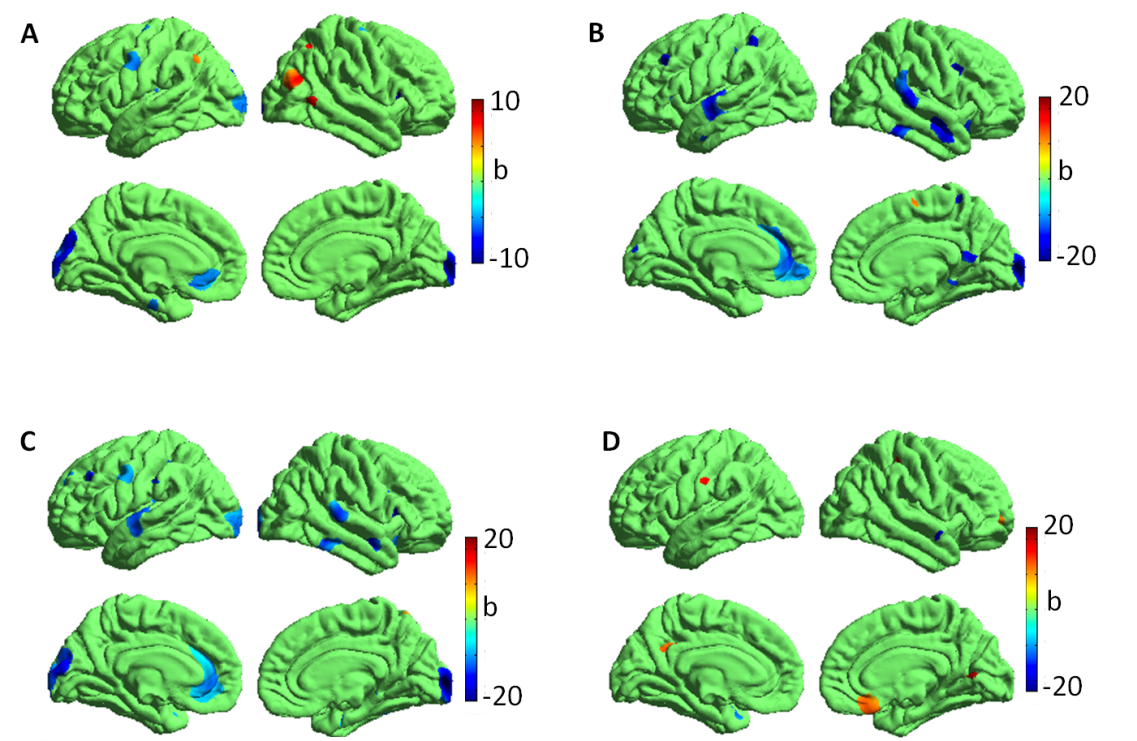


**Fig. S3.** The regression analysis of cortical thickness and clinical variables in OCD patient group. Age, gender, and IQ are entered as covariates. Color indicates the regression coefficient of cortical thickness with clinical scores. Subfigures (A-D) denote the results on 4 different clinical scores: (A) Obsessive subtotal scores on the Y-BOCS; (B) Compulsive subtotal scores on the Y-BOCS; (C) Total scores of Y-BOCS; (D) Total scores of OCI-R.
