# Supplementary material for: Structural and Diffusion Property Alterations in Unaffected Siblings of Patients with Obsessive-Compulsive Disorder
Source: PLoS One. 2014 Jan 28;9(1):e85663. doi: 10.1371/journal.pone.0085663 (PMC3904847; doi:10.1371/journal.pone.0085663)
Supplement: Table S1 — Treatment details of OCD patients. (ZIP) [file pone.0085663.s004.zip]

**Table S1** Treatment details of OCD patients.

| **Treatment** | **Number of cases** | | **Average dosage (mg)** | |
| --- | --- | --- | --- | --- |
| citalopram | | 2 | | 40 |
| clomipramine | | 2 | | 100 |
| fluoxetine | | 3 | | 46.7 |
| fluvoxamine | | 3 | | 250 |
| mirtazapine | | 2 | | 30 |
| paroxetine | | 6 | | 60 |
| sertraline | | 3 | | 162.5 |
| venlafaxine | | 2 | | 225 |
| paroxetine + sodium valproate | | 3 | | 40 + 666.7 |
| paroxetine + quetiapine | | 2 | | 40 + 200 |
| paroxetine + lithium carbonate | | 2 | | 40 + 500 |
