# Supplementary material for: Structural and Diffusion Property Alterations in Unaffected Siblings of Patients with Obsessive-Compulsive Disorder
Source: PLoS One. 2014 Jan 28;9(1):e85663. doi: 10.1371/journal.pone.0085663 (PMC3904847; doi:10.1371/journal.pone.0085663)
Supplement: Table S2 — Inter-correlations between the anatomical measurements. The correlation coefficients between each pair of mean FA, fiber-quantity (FQ) and mean cortical-thickness (CT) values from each of the 5 ROIs are shown below. (ZIP) [file pone.0085663.s005.zip]

**Table S2** Inter-correlations between the anatomical measurements. The correlation coefficients between each pair of mean FA, fiber-quantity (FQ) and mean cortical-thickness (CT) values from each of the 5 ROIs are shown below.

| Starting points used for fiber tracking | FA - FQ | FA - CT | FQ - CT |
| --- | --- | --- | --- |
| L arcuate fibers near superior parietal lobule | **0.35^*^** | **0.33^*^** | **0.27^*^** |
| R cingulum near corpus callosum-body | 0.13 | -0.04 | **0.46^*^ ^*^** |
| L cingulum near corpus callosum-genu | **0.23^*^** | -0.06 | 0.55 |
| L arcuate fibers near precentral gyurs | 0.10 | 0.19 | **0.42^*^ ^*^ ^*^** |
| R ILF near superior temporal gyrus | 0.18 | 0.12 | 0.70 |

ILF: inferior longitudinal fasciculus; L: left; R: right.

^*^ *P* < 0.05

^**^ *P* < 0.01

^***^ *P* < 0.001
